# Preparation of Multifunctional Fe@Au Core-Shell Nanoparticles with Surface Grafting as a Potential Treatment for Magnetic Hyperthermia

**DOI:** 10.3390/ma7020653

**Published:** 2014-01-24

**Authors:** Ren-Jei Chung, Hui-Ting Shih

**Affiliations:** Department of Chemical Engineering and Biotechnology, National Taipei University of Technology (Taipei Tech), Taipei 10608, Taiwan; E-Mail: huiting7931@yahoo.com.tw

**Keywords:** methotrexate, indocyanine green, core-shell Fe@Au-PSMA-ICG/MTX nanoparticles, magnetic hyperthermia treatment, *in vitro* studies

## Abstract

Iron core gold shell nanoparticles grafted with Methotrexate (MTX) and indocyanine green (ICG) were synthesized for the first time in this study, and preliminarily evaluated for their potential in magnetic hyperthermia treatment. The core-shell Fe@Au nanoparticles were prepared via the microemulsion process and then grafted with MTX and ICG using hydrolyzed poly(styrene-alt-maleic acid) (PSMA) to obtain core-shell Fe@Au-PSMA-ICG/MTX nanoparticles. MTX is an anti-cancer therapeutic, and ICG is a fluorescent dye. XRD, TEM, FTIR and UV-Vis spectrometry were performed to characterize the nanoparticles. The data indicated that the average size of the nanoparticles was 6.4 ± 09 nm and that the Au coating protected the Fe core from oxidation. MTX and ICG were successfully grafted onto the surface of the nanoparticles. Under exposure to high frequency induction waves, the superparamagnetic nanoparticles elevated the temperature of a solution in a few minutes, which suggested the potential for an application in magnetic hyperthermia treatment. The *in vitro* studies verified that the nanoparticles were biocompatible; nonetheless, the Fe@Au-PSMA-ICG/MTX nanoparticles killed cancer cells (Hep-G2) via the magnetic hyperthermia mechanism and the release of MTX.

## 1. Introduction

In recent decades, nanotechnology has been one of the most popular research areas. Nanomaterials with different geometries in zero- to three-dimensional scales have been synthesized, including nano powders, nano fibers, nano films and nano bulks [1,2]. Nanoparticles are the most basic and diverse of these structures. Through surface grafting, nanoparticles made of the same component can exhibit different properties and have tailor-made functions [3]. Metallic nanoparticles have been studied and applied in the optical, catalytic, magnetic and biomedical research fields [4–7]. Magnetic nanoparticles have the properties of quantum effects, a large surface area and diverse magnetic properties compared with bulk material. When the particle size approaches a single magnetic domain, superparamagnetic characteristics are observed. These superparamagnetic nanoparticles can be utilized for the controlled release of medicine, magnetic hyperthermia treatment and bio-imaging [8–10].

Gold, which has been treasured and utilized by people throughout history, has biomedical applications because of its excellent biocompatibility and numerous advantages, including *in vivo* stability, facile preparation, convenient grafting using thiol bonds and a controllable reduction process [11]. In addition to gold nanoparticles, gold is usually coated on other substances to achieve the core-shell structure with the benefit of compromising properties [12,13]. Gold shells help extend the period of stability and provide protection against reactions [14]. Nanoparticles a few nanometers in size composed of Fe, Fe_3_O_4_, core-shell Fe_3_O_4_@Au and Fe@Au have been reported to be superparamagnetic and have been studied for magnetic hyperthermia applications [15–17]. Because malignant cells lack the appropriate heat shock response, cancer cells die prior to normal cells when the tissue temperature is heated above 41°C. Magnetic hyperthermia treatments exploit this mechanism to selectively eliminate malignant cells [18].

Methotrexate (MTX), a folic acid antagonist, is an effective anti-cancer therapeutic and immunologic agent [19]. Folic acid is an important precursor for metabolism. MTX targets and treats blood cancers, lymphoma, head and neck cancer and rheumatoid arthritis. Indocyanine green (ICG) is a fluorescent dye with an absorption peak between 800 and 810 nm and an emission approximately 835 nm. ICG can pinpoint the location of liver cancer and becomes visible in response to excitation with near infrared light [20,21].

**Figure 1.**
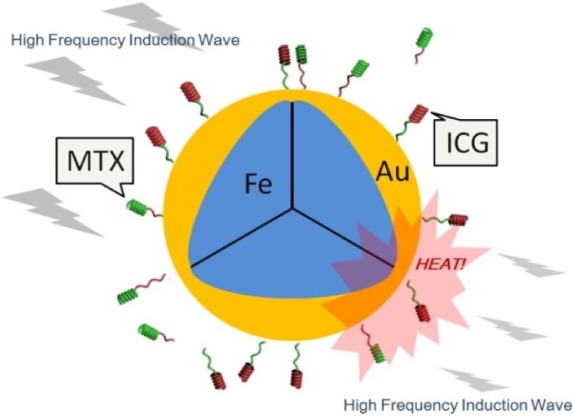
A schematic of the Fe@Au-PSMA-ICG/MTX nanoparticles.

The aim of this study, as shown in Figure 1, was to prepare core-shell Fe@Au nanoparticles via the microemulsion process and then graft the nanoparticles with MTX and ICG using hydrolyzed poly(styrene-alt-maleic acid) (PSMA) to obtain Fe@Au-PSMA-ICG/MTX nanoparticles. We aimed to develop a multifunctional system that was biocompatible, magnetic-induced, targeted cancer cells, labeled specifically and had potential as a magnetic hyperthermia treatment.

## 2. Results and Discussion

**Figure 2.**
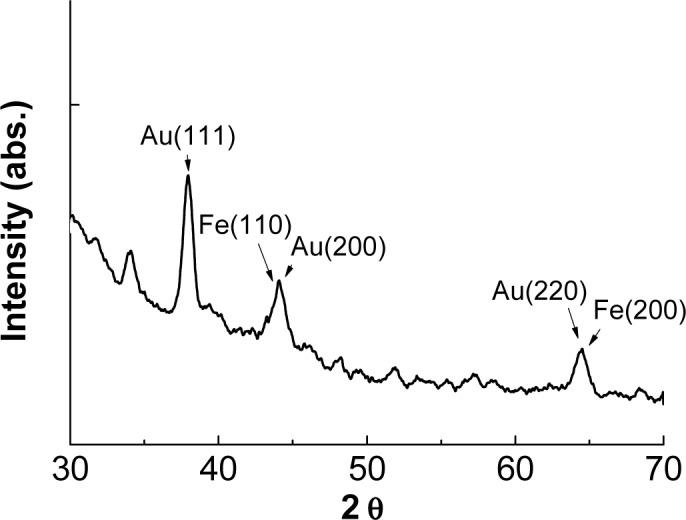
X-ray powder diffraction (XRD) pattern of the prepared nanoparticles.

**Figure 3.**
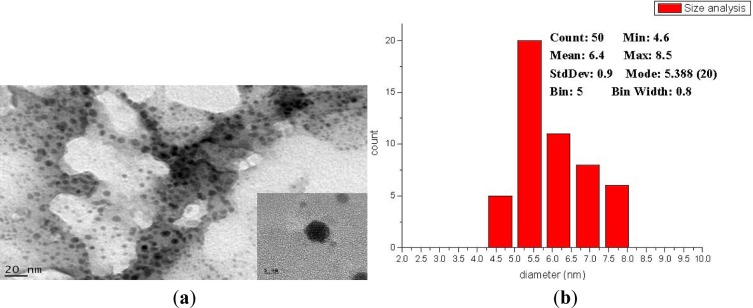
(**a**) Transmission Electron Microscope (TEM) image of the nanoparticles; (**b**) Image J analysis of particle size.

Figure 2 illustrates the XRD pattern of the nanoparticles. The 2θ peaks at 37.97°, 44.32° and 64.37° corresponded to the (111), (200) and (220) planes of fcc gold; the 2θ angles at 43.85° and 64.71° corresponded to the (110) and (200) planes of bcc iron [7]. The XRD results indicated that the Au coating protected the Fe core from oxidation and growth. Figure 3a presents the TEM micrographs and high resolution image of the prepared nanoparticles. According to the TEM images, the nanoparticles were well separated with around 1 nm Au coating. The Au coating helped the suspension and prevented it from aggregating. The particle size was analyzed using ImageJ software (National Institutes of Health) [7], and the results indicated that the average particle size was 6.4 ± 0.9 nm.

By examining the surface characteristics of the particles in a pH of approximately 7, the zeta potential of the Fe@Au nanoparticles was determined to be −19.8 mV. After surface modification, the zeta potential of the Fe@Au-PSMA nanoparticles was −42.8 mV. The massive decrease in surface charge resulted from the successful coating of poly(styrene-alt-maleic acid). After the final grafting, the zeta potential of the Fe@Au-PSMA-ICG/MTX nanoparticles was −23.6 mV. The negative surface charge helped the nanoparticles stay in suspension in water.

**Figure 4.**
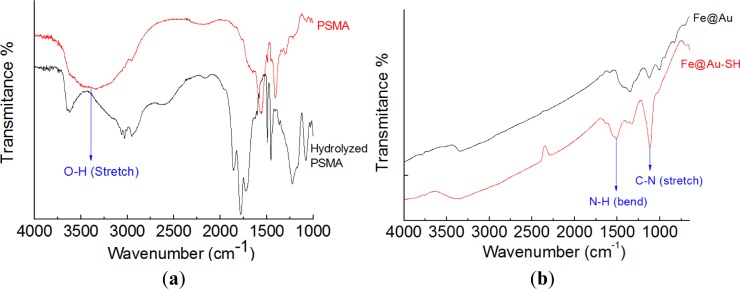

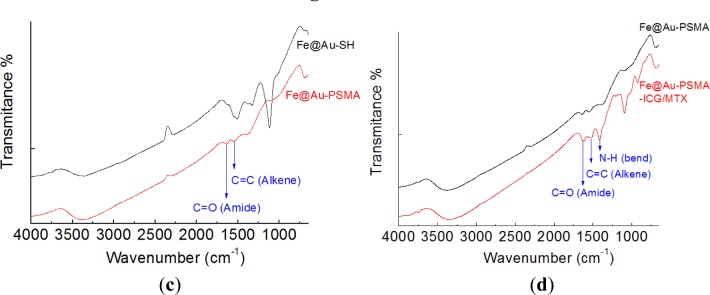
Fourier Transfer Infrared (FTIR) profiles of (**a**) poly(styrene-alt-maleic acid) (PSMA) *vs*. hydrolyzed PSMA; (**b**) Fe@Au *vs*. Fe@Au-SH; (**c**) Fe@Au-SH *vs*. Fe@Au-PSMA; and (**d**) Fe@Au-PSMA *vs*. Fe@Au-PSMA-ICG/MTX.

**Figure 5.**
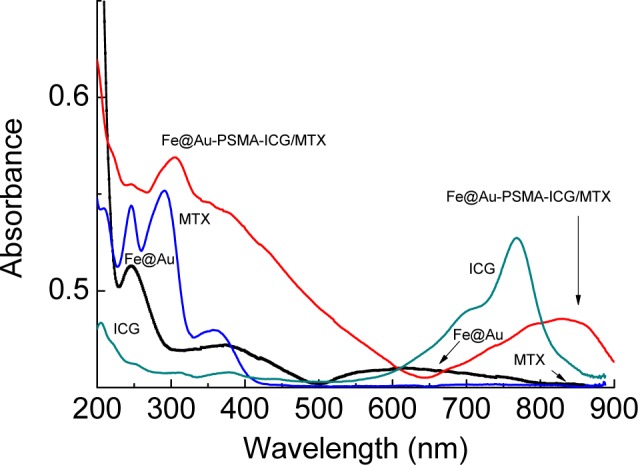
UV-Vis spectra of indocyanine green (ICG), methotrexate (MTX), Fe@Au and Fe@Au-PSMA-ICG/MTX.

Figure 4 illustrates the FTIR profiles that were utilized to monitor the preparation steps. After the open ring reaction, the obvious absorption at 3421 cm^−1^, corresponding to O-H stretch, indicated the successful preparation of hydrolyzed PSMA (Figure 4a). The peaks at 1350–1000 cm^−1^ (C-N stretch) and 1640–1450 cm^−1^ (N-H bend) provided evidence that 2-aminoethanethiol was successfully attached to Fe@Au to form Fe@Au-SH (Figure 4b). After the PSMA coating, peaks corresponding to C=C (alkene, 1680–1600 cm^−1^) and C=O (amide, 1680–1630 cm^−1^) were observed (Figure 4c). Figure 4d illustrates the FTIR profile of Fe@Au-PSMA-ICG/MTX. Several specific peaks corresponding to PSMA, ICG and MTX are indicated, including C=C (alkene, 1680–1600 cm^−1^), C=O (amide, 1680–1630 cm^−1^), C-N stretch (1350–1000 cm^−1^) and N-H bend (1640–1450 cm^−1^). UV-Vis absorption spectrometry was utilized to verify the optical properties of the nanoparticles and the bonding of ICG and MTX. Figure 5 presents the UV-Vis spectra for ICG, MTX, Fe@Au and Fe@Au-PSMA-ICG/MTX. The specific absorption peak of ICG is approximately 780 nm, and that of MTX is approximately 300 nm. The spectrum of the Fe@Au-ICG/MTX nanoparticles confirmed the successful surface grafting.

**Figure 6.**
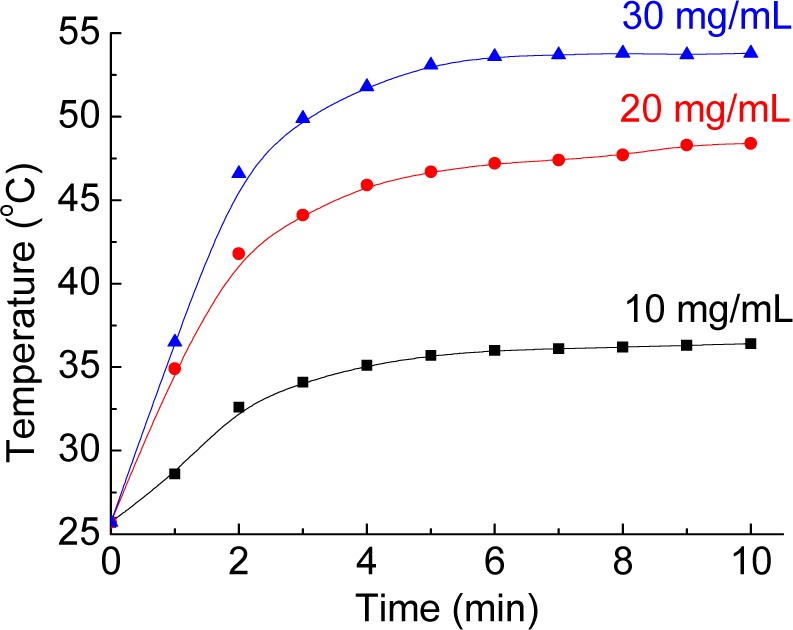
The temperature elevation tests of different nanoparticle concentrations in water.

Figure 6 depicts the profiles of the temperature elevation tests of different nanoparticle concentrations in water. Under exposure to HFIW, the nanoparticle solution was efficiently heated. The temperature rose rapidly in 5 min and then stabilized. The results suggested a trend towards concentration-dependence. At 10 mg/mL, the temperature increased by 10.7°C in 10 min; at 20 mg/mL, it increased by 22.7°C, and at 30 mg/mL, it increased by 28.1°C. Considering the clinical application for hyperthermia, the temperature would be expected to rise from 37°C to 42°C. The efficiency of the Fe@Au-ICG/MTX nanoparticles is believed to be sufficient.

**Figure 7.**
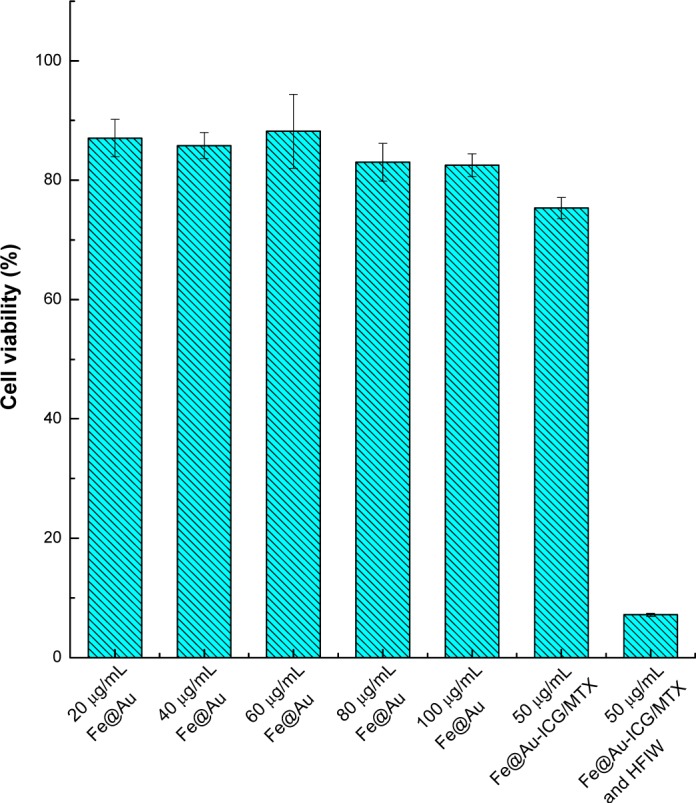
The WST-8 assay in Hep-G2 cells treated with different concentrations and compositions of nanoparticles.

The results of the *in vitro* experiments are presented in Figure 7. The cell viability was tested using WST-8 Cell Proliferation Assay Kit (CAS number 193149-74-5) and calculated relative to the blank medium (100%). The cell viabilities were all above 80% after treatment with Fe@Au nanoparticles at concentrations of 20, 40, 60, 80 or 100 μg/mL. Fe@Au-PSMA-ICG/MTX nanoparticles at 50 μg/mL were selected to investigate the magnetic hyperthermia efficiency, and the cell viability was approximately 75%. Because MTX is a folic acid antagonist and therefore is an anti-cancer treatment, MTX release from the nanoparticles would result in cell death. However, compared with free MTX, carriers or vectors have been reported to efficiently reduce the cytotoxicity and achieve controlled release [7].

After treatment with 50 μg/mL Fe@Au-PSMA-ICG/MTX and a 10-min exposure to HFIW, the cell viability decreased to less than 10%. The results indicated that the Fe@Au-PSMA-ICG/MTX nanoparticles efficiently killed the cancer cells via the magnetic hyperthermia mechanism and the release of MTX.

## 3. Experimental Section

### 3.1. Preparation of Fe@Au Nanoparticles via the Microemulsion Method

To prepare the Fe@Au nanoparticles, a degassed octane aqueous solution served as the oil phase, 1-Butanol functioned as a surfactant and cetyltrimethylammonium bromide (CTAB) was utilized as the self-assembly template. Solutions of 0.2 M FeSO_4_, 0.5 M NaBH_4_, 0.2 M HAuCl_4_ and 0.8 M NaBH_4_ were added separately into the CTAB/1-Butanol/isooctane solution to produce solutions 1, 2, 3 and 4, respectively. The final concentrations of these four microemulsion precursors are presented in Table 1. Equal volumes of solutions 1 and 2 were thoroughly mixed to obtain pure iron nanoparticles. Solutions 3 and 4 were added into the iron nanoparticle solution in sequence, thus synthesizing the Fe@Au nanoparticles. The Fe@Au nanoparticles were purified using alcohol and collected using an NdFeB magnet.

### 3.2. Preparation of Fe@Au-PSMA-ICG/MTX via Surface Modification

Fe@Au nanoparticles (20 mg) and 50 mg 2-aminoethanethiol were mixed in 5 mL of DI water at 45°C with ultrasonication for 4 h to form the Au-S bonds. The functionalized nanoparticles (Fe@Au-SH) were purified using alcohol, collected with an NdFeB magnet and freeze dried.

Hydrolyzed poly(styrene-alt-maleic acid) (PSMA) was prepared by treating PSMA in 1 M NaOH for 18 h. Hydrolyzed PSMA (1 mg) was dissolved in 4.9 mL of 0.03 M phosphate buffered saline (PBS) with a pH between 5.42 and 7.05, and subsequently 0.1 mL 1-ethyl-3-(3-dimethyl aminopropyl)-carbodiimide hydrochloride (EDC) and 10 mg Fe@Au-SH nanoparticles were added under ultrasonication for 30 min to produce Fe@Au-PSMA nanoparticles.

Finally, Fe@Au-PSMA nanoparticles, ICG and MTX were mixed in the dark at a weight ratio of 5:1:1 under ultrasonication for 30 min and then stirred for 4 h. Fe@Au-PSMA-ICG/MTX nanoparticles (Figure 1) were collected using an NdFeB magnet, freeze dried and stored in the dark.

### 3.3. Characteristics of the Nanoparticles

The crystalline phases of the nanoparticles were examined by X-ray powder diffraction (XRD, XRD-6000, Shimadzu, Japan) using CuKα radiation (λ = 1.5406 nm). A transmission electron microscope (TEM, JEM-1230, JEOL, Japan) was utilized to observe the geometries of the nanoparticles. The zeta potential of the nanoparticles was measured with a Dynamic Light Scattering Instrument (DLS, Nano-ZS90, Malvern, UK). A Fourier Transfer Infrared Instrument (FTIR, PerkinElmer Instruments Spectrum 2000, UK) was utilized to investigate the chemical composition of the materials. The optical properties were analyzed by UV-Vis absorption spectrometry (Jasco V655 UV/visible spectrophotometer, UK).

A temperature elevation test was performed to examine the efficiency of the magnetic hyperthermia treatment. Nanoparticle solutions (10, 20 and 30 mg/mL) were exposed to a high frequency induction wave (HFIW, 1.1 MHz) via high frequency induction heating units (Power Cube 32, CEIA, Italy), and the temperature was monitored *in situ* using an optical fiber thermometer.

### 3.4. *In Vitro* Cytotoxicity Assay

Human Hep-G2 hepatoma cells were cultured for *in vitro* studies. Cells (10^4^ per well) were seeded in 96-well plates for 24 h. Before the tests, the purified nanoparticles were sterilized with UV light for 30 min; they were subsequently added to cell culture medium (Dulbecco’s Modified Eagle’s medium, DMEM, Gibco, USA) containing 10% fetal bovine serum (FBS, Invitrogen, Carlsbad, CA, USA), 0.37% w/v NaHCO_3_, 100 U/mL penicillin, 100 μg/mL streptomycin and 0.03% w/v glutamic acid). The nanoparticle suspension in medium was added to the cell cultures. After 24 h, the medium was removed, and 10 μL WST-8 Cell Proliferation Assay Kit solution was added for 4 h. A microplate reader (Infinite M200, TECAN, Switzerland) was utilized to measure the absorbance at 450 nm, and the cell viability was calculated. The Fe@Au nanoparticles were tested at 20, 40, 60, 80 and 100 μg/mL. Medium without nanoparticles was utilized as the control group.

To perform the *in vitro* magnetic hyperthermia test of the Fe@Au-PSMA-ICG/MTX nanoparticles, the cells were treated with 50 μg/mL nanoparticles for 24 h. The cultures were washed using adequate PBS, exposed to HFIW for 10 min and provided with medium for 24 h. The cell viability was evaluated via the WST-8 test.

## 4. Conclusions

Core-shell Fe@Au nanoparticles prepared via the microemulsion process with surface grafting of MTX and ICG have been synthesized for the first time in this study. MTX is an anti-cancer therapeutic, and ICG is a fluorescent dye. The XRD and TEM results revealed that the average size of the nanoparticles was 6.4 ± 0.9 nm and that the Au coating protected the Fe core from oxidation. The FTIR and UV-Vis spectrometry characterizations confirmed the successful surface grafting. After exposure to HFIW, the superparamagnetic nanoparticles elevated the solution temperature in a few minutes. The *in vitro* experiments verified that the nanoparticles were biocompatible; nonetheless, the Fe@Au-PSMA-ICG/MTX nanoparticles killed cancer cells via the magnetic hyperthermia mechanism and the release of MTX.

## Figures and Tables

**Table 1.** Final concentrations in the precursor solutions (wt%).

Reagent							
Symbol	0.2 M FeSO_4_	0.5 M NaBH_4_	CTAB	1-Butanol	Isooctane	0.2 M HAuCl_4_	0.8 M NaBH_4_
Solution 1	21%	–	18%	53%	8%	–	–
Solution 2	–	21%	–	–	–	–	–
Solution 3	*–*	–	14.5%	57.8%	10.4%	17.3%	–
Solution 4	–	–	–	–	–	–	17.3%
